# Dentistry as a "Fine Art." No. 2

**Published:** 1867-07

**Authors:** Norman W. Kingsley

**Affiliations:** Professor of Dental Art and Mechanism, in the New York College of Dentistry.


(To be Continued.)

---

### ARTICLE IV.

### *Dentistry as a "Fine Art."* *No.* 2.

By Norman W. Kingsley, Professor of Dental Art and Mechanism, in the New York College of Dentistry.

If Dentistry as we have claimed in a former article is a speciality of one of the Fine Arts, it must be subject to the same general rules.

We will endeavor to make this application, and in so doing shall quote to some extent from authors upon Art; showing that for such purposes, all rules that govern the

Fine Arts are not limited in their application to one of its forms, but that everything that relates to ideality—the distinguishing characteristic of a fine art—is equally applicable to all, only in a different degree.*

Dentistry as an art, we have stated is but a department of Sculpture.

A more intimate acquaintance with this, its parent, will will both interest and instruct us.

There is probably a more prevalent ignorance of the Mechanism of Sculpture than of that of any other art.

In Poetry, Music and Painting, any mind of ordinary cultivation has a tolerably correct idea of the means whereby the end is accomplished.

In Painting, the knowledge that a sketch is made, and the colors laid on with a brush and harmonized and blended until the effect is produced, comes almost intuitively at the first sight of the result. But in Sculpture the general knowledge of the means employed to accomplish the end, is exceedingly crude. The respect for the art, very often consists in an astonishment at the labor that must have been spent in cutting the marble, and the difficulty that must have attended the production of such delicate forms from such adverse material.

It is but a short time since that in an essay which was read before a Western Dental Society, the writer used for an illustration a reference to the art of Sculpture, which conveyed the prevailing impression that the ideal of the artist existed in his mind until by repeated strokes with hammer and chisel, and weary months of labor in waiting and watching, he was at last rewarded by seeing slowly emerge from its rock-bound covering, the ideal of his imagination.

The illustration was very pleasingly worded, and probably, on the majority of his hearers, made the same impression as if it had been true. Let us visit a sculptor's studio

*No attempt will be made to give the names of authors, as in many instances the names are unknown to the writer.

and become acquainted with his machinery and *modus operandi*.

What do we find ? A plain and not altogether cheerful apartment, with coarse walls of a neutral tint ; a window high up with a northern exposure, the light falling on the objects below at an angle varying more or less from 45 degrees ; a tub full of moist clay—common, ordinary *earthy clay :* half a dozen sticks of different forms, the size of a pen-holder, and—that is all.

This is the sum total of an artist's requisites, no hammers, chisels, machinery or marble ; all *that* belongs to the stone-cutter's trade, and not to the artist.

With these simple appliances he produces those works which have astonished and will astonish the world until the end of time.*

Out of the plastic clay he fashions with skillful and nimble fingers the form that has dwelt in his imagination, not by wearisome days of labor with still more wearisome nights of waiting to see the result, but with a rapidity surpassing description does the soft and pliant clay yield to his magic touch, and out of a shapeless mass in a single day often, will appear form and comeliness.

Now with a servant so willing to obey his mandates is he able to fix almost immediately, the thoughts that have cost him months of preparation.

Every effort of the brain in the production of a statue is spent upon this clay model. It is this which he studies, and as he knows that *every variation of the form changes the expression*, and *that expression is the key to the character*, so does he bend with all earnestness to every detail ; building up here and depressing there, swelling out this muscle and

*It is also a very prevalent error that the production of a portrait bust is the result of a cast taken of the face.

While such casts are sometimes taken of deceased persons, no true artist makes any further use of them than as works of reference.

Usually the bust is modeled in the clay from sittings, and no plaster or other material comes in contact with the face.

relaxing that, until in satisfaction his work is consummated.

This model in clay is the end of the *artist's* labor, and the *end* in more senses than one it might be said, as the very next step of the process involves its destruction.

The mechanic now takes it out of his hands, and every succeeding operation until it appears the finished marble is only one of mechanism. First then comes the moulder in plaster, and this exquisite image of the artist's creation, beautiful and life-like to dwell upon even in the clay, is by him buried out of sight in a winding sheet of plaster; and in its removal from this mould it is destroyed; while in its stead, ultimately, appears a duplicate, in dead and lifeless plaster.

No one who has not admired the original as it left the artist's hands, full of the warmth of nature which only the clay can give, and then seen substituted for it this cold and inanimate body, can fully realize Thornwalsden's charming simile:

"The clay represents the Life, the plaster the Death, and the marble the Resurrection." And this resurrection is indeed that of a glorified body; in form, a copy of the original, but in appearance not of the earth, earthy: and all these successive steps are purely mechanical.

The cutter of stone, with chisel, callipers and compasses, by taking careful measurements, will accomplish the whole to perfection, even adding to its beauty, in finish, without the brain to conceive a single portion of it.

Let us analyze the knowledge, and the education necessary to acquire it, which so remarkably distinguishes here the artist from the mechanic.

The true artist "is distinguished not so much by uncommon powers of mind, as by an uncommon combination of powers designated by the name of genius; free imagination, fine sentiment, both moral and intellectual, clear discrimination, philosophical reasoning, and sound judgement, are all essential to the best productions of ideal art.

With imagination, perception, and judgment, an artist may produce works of a high order, but if his mind is deficient in philosophical power, he can be neither a great poet, painter or sculptor, for true philosophy is the foundation of all art."

There must also exist a love for and an appreciation of the creations of the Divine Artist, and an enthusiasm in the contemplation of them.

The seed of this love is born in the man, its full developement is the result of cultivation.

No man can ever hope to excel in any art for which he has no love, his labor will degenerate into drudgery, and his enthusiasm into disgust. But it is not alone the possession of natural talents, cultivated tastes or enthusiastic love, which brings success.

The acquisition of much scientific knowledge is also essential. The Sculptor and Painter must "understand the Science of Anatomy, or he cannot represent his forms correctly; the Science of optics, on which depends his light and shade, perspective and color, the Science of mathematics, or he cannot apply these laws—the Science of chemistry, that he may know the nature of his materials.

"He must also understand the laws of gravity,—the laws of harmony and beauty---the laws of expression, both in countenance and attitude; and finally the laws of the human mind, to which his work is addressed."

"In addition to these specific acquirements he must have an extensive general knowledge, and a skill of hand acquired only by years of practice. He may be master of all the Sciences, and yet without this last accomplishment he cannot even make a copy, much less execute an original design."

We have placed Anatomy at the head of the Sciences, because it should form the corner-stone of the education of the Artist in scientific knowledge; the form, the locality, and the uses of every bone, joint, and muscle must be as thoroughly in his mind, as in the mind of the surgeon.

The effect of the movement of each joint and muscle must be clearly comprehended; every variation of which, especially with the muscles of the face, gives a new expression. He therefore who would become master of the art of producing an expression which shall be in harmony with the character, must become acquainted with its physical causes.

The most accomplished artists possess this acquaintance with Anatomy. Michael Angelo studied Anatomy twelve years. He was not only a Painter, but equally a Sculptor and an Architect, and it was this thorough knowledge thus acquired of the grammar of Art, that gave him such preeminence. Some of his sketches show that his practice was first to draw the bones, and then fill out the figure upon that skeleton.

By his perfect familiarity with every muscle in the human frame, he knew precisely which one should be brought into action, to express the passion or emotion that he wished to delineate.*

To this comprehension of the physical structure of the human frame,—which can be acquired in the same manner as that of any other exact science,—must be added some knowledge of Physiognomy; which can only come in its fulness from long continued observation of the infinite variety of faces which we are continually meeting. The harmonious relations of one feature with another, must be so fully comprehended, that it will be possible to restore a lost part, in all its perfection, by a knowledge of what is demanded by those features remaining. When the celebra-

*J. Quincy A. Ward, the best Classical Sculptor now living that America has produced, makes this perfect knowledge of Anatomy one of the main secrets of his success. In the statue of Commodore Perry, a figure nearly eight feet high, which he is now modeling, his practice shows the application of these principles.

Although the statue was to be draped from the chin to the extremities in the costume of the character, the Artist modeled the figure, nude; carrying out every detail of the external Anatomy perfectly, and subsequently proceeded to clothe it in suitable drapery.

ted statue, the Apollo Belvedere, was discovered in the ruins of ancient Antium, one of the hands and other portions of the body were mutilated. The restoration was made by Montorsali, but so bunglingly accomplished, that it has never received the approval of Artists. Nevertheless the statue is regarded as the most beautiful figure of its kind in existence. In like manner the features of the face must present no incongruity ; the one with another, and the whole with the character.

It is claimed by Physiognomists that the true character may be read in the countenance; but whether Physiognomy is yet reduced to so exact a Science as this statement would indicate, certain it is that the natural characteristics of mankind are very strongly marked in the face, and so generally is this accepted as a truth, that first impressions received, are acted upon with a very strong faith in their correctness.

The nature of an individual is very often refined by external influences, until the face is no longer a complete index to it; but even then the physical conformation is slowly modified and ultimately harmonizes with it.

The growth of nations in christianity and civilization abundantly proves this fact. Nations whose early history shows them to have been but very little above the brutes, in their gross sensualism and savage ferocity, and whose countenances bore the marks of their natures, have as they advanced in refinement, developed also, into beauty and comeliness.

Individual cases like this often take place in a single generation, and instances are not rare which are within the knowledge of any observer It is said that the life of Socrates was a continual struggle against his natural propensities, and if the busts that have been preserved of him are authentic portraits, we may well believe the statement. The face is certainly not one we should picture as belonging to such a character.

From accidental causes also, the face may become deformed, by the absorption of bony processes; the wasting of flesh and muscles, etc., until an expression will result which does not indicate the real nature of the individual. Portrait Sculpture is only redeemed from mechanism, and made a fine art, by this infusion of the moral character into the expression of the face.

If the artist fails to appreciate the noble and distinguishing traits of his sitter, and thus depict them upon the countenance, his work has not been worthy the labor spent upon it. He has but given us a copy of the form, but no clue whatever to the soul which animates it. All artists and physiognomists agree that the mouth presents a greater variety of expressions than any other feature. In Portrait Sculpture the mouth is the feature of all others for denoting expressions.

Neither the eyes, nose, forehead, ears or chin, or all combined have the power of conveying that of which the mouth is capable.

It speaks, without utterance, of every emotion of the the heart, love, anger, pride, scorn and contempt equally with joy and sorrow, have their insignia stamped upon the mouth.

These changes are so rapid and their continuance so evanescent, that the phrase "catch the expression" is often used with but little idea of its full signification.

It is this which makes portraits by a true artist invaluable as contrasted with many of the productions of the photographer. The one catches the fleeting expression of the soul and transfixes it for all time, the camera of the photographer but pictures the unanimated features.

"All parts of the face, doubtless have their fixed relations to each other and to the character of the person to whom the face belongs. But there is one feature and especially one part of that feature, which more than any other facial sign reveals the nature of the individual. The feature is the *mouth,* and the portion referred to is the *corner*. A circle of half an inch radius, having its centre at the junction of the two lips will indicate the chief focus of the expression."—(Dr. Holmes.)

"In cheerful emotions as laughter, smiling, etc., the angles are pulled upwards. In fear, pride, hatred, revenge, disgust, contempt, consciousness of power, the corners of the mouth are drawn downwards. The union of so many muscles at the angles of the lips, produces that fullness about the mouth remarkable in those who are thin and muscular. In the child or youth whose face is plump, they make the dimple in the cheek.

The orbicularis is the opponent of all the muscles which are concentrated from various points to the lips; and it is by the successive action and relaxation of these antagonistic muscles that so much and so varied expression is given to the mouth.

This circular muscle, which has no origin and goes entirely around the mouth, is effected in various emotions.

It tremblingly yields to the superior force of its counteracting muscle, both in joy and in grief. It relaxes pleasantly in smiling. It is drawn down more powerfully by its opponent muscles in weeping. This is the largest and strongest muscle of the face; it antagonizes all the rest, shuts the mouth, and from an opening as wide as the mouth can require, it shuts it at pleasure, so closely as to retain the breath against all the force of the lungs.

It is the true antagonist of all the other muscles; yet it acts mutually with them, in opening and shutting the mouth.

The bones determine the general form of the face; one great muscle, the masseter, gives the rounding of the cheek; the rest are delicate and moveable muscles, and the character of the face centres around the mouth and nostrils where those muscles converge.

A thin and delicate face gains in expression where the cheek is hollow, and at the angle of the mouth where the lines are strong. In a full face, these lines are obliterated, and the delicate turnings of thought and feeling are lost. All but the more violent expressions of passion are buried in the mass.

The great lines of character are the lines of the Zygomatic muscle, coming from above, and of the triangular muscle, coming from the chin, and the moving point towards which they all act, is the corner of the mouth.

In cheerful emotions they all rise towards the eye, which becomes full and distended.

In the depressing passions the features sink, the eye is languid, and the whole countenance has a serious, thoughtful cast; still the corner of the mouth is the central point of all these changes.

The corners of the mouth are continually supported by the action of the Zygomatic muscles.

They are raised in smiling so as to form the dimple; in laughter still higher, so as to swell the cheek, wrinkle the eyelids, and compress the eyes until the tears begin to flow. The corner of the mouth that is thus raised in laughter, is distorted in pride and drawn backward in rage, drops lower in grief, and in palsy falls quite down.

These various movements around the angle of the mouth are the chief indications of passion in the face, requiring careful observation for their full comprehension, and it must have already become apparent that this knowledge is of equally vital importance to him who would succeed in the art of Dentistry, as in the arts of sculpture and painting.

The "consummation of excellence is not attained until the artist has so mastered the rules that guide his practice that, in the execution of his work, he conceals all evidence of labor. It is this rare attainment, the art of concealing art, that leads to the erroneous belief in the inspiration of genius, that not only furnishes ideas to the gifted, but the mechanism essential to their right expression."

Herein is Dentistry a fine art; an "ideal art" in its full signification.

In the loss of the teeth, the absorption of the processes and the wasting away of the muscles and tissues as we have seen, the greatest possible detriment is caused to the expression of the human countenance.

The complete restoration of this feature, with all its power of expression, by art; art so consummate in the selection, arrangement and adaptation of its means as to defy detection, is the crowning glory of Dentistry *as an art.*

